# Comparative quantitative monitoring of rabbit haemorrhagic disease viruses in rabbit kittens

**DOI:** 10.1186/1743-422X-11-109

**Published:** 2014-06-09

**Authors:** Markus Matthaei, Peter J Kerr, Andrew J Read, Paul Hick, Stephanie Haboury, John D Wright, Tanja Strive

**Affiliations:** 1 Commonwealth Scientific and Industrial Research Organisation – Ecosystem Sciences, ACT 2601 Black Mountain, Australia; 2 Elizabeth Macarthur Agricultural Institute, Woodbridge Rd, 2568 Menangle, NSW, Australia; 3 Invasive Animals Cooperative Research Centre, University of Canberra, Canberra ACT 2601, Australia; 4 CSIRO – Ecosystem Sciences, GPO Box 1700, Canberra ACT 2600, Australia

**Keywords:** RHDVa, *Oryctolagus cuniculus*, Time course, Resistance, RHD, Invasive species, Biological pest control, Calicivirus

## Abstract

**Background:**

Only one strain (the Czech CAPM-v351) of rabbit haemorrhagic disease virus (RHDV) has been released in Australia and New Zealand to control pest populations of the European rabbit *O. cuniculus*. Antigenic variants of RHDV known as RHDVa strains are reportedly replacing RHDV strains in other parts of the world, and Australia is currently investigating the usefulness of RHDVa to complement rabbit biocontrol efforts in Australia and New Zealand. RHDV efficiently kills adult rabbits but not rabbit kittens, which are more resistant to RHD the younger they are and which may carry the virus without signs of disease for prolonged periods. These different infection patterns in young rabbits may significantly influence RHDV epidemiology in the field and hence attempts to control rabbit numbers.

**Methods:**

We quantified RHDV replication and shedding in 4–5 week old rabbits using quantitative real time PCR to assess their potential to shape RHDV epidemiology by shedding and transmitting virus. We further compared RHDV-v351 with an antigenic variant strain of RHDVa in kittens that is currently being considered as a potential RHDV strain for future release to improve rabbit biocontrol in Australia.

**Results:**

Kittens were susceptible to infection with virus doses as low as 10 ID_50_. Virus growth, shedding and transmission after RHDVa infection was found to be comparable or non-significantly lower compared to RHDV. Virus replication and shedding was observed in all kittens infected, but was low in comparison to adult rabbits. Both viruses were shed and transmitted to bystander rabbits. While blood titres indicated that 4–5 week old kittens mostly clear the infection even in the absence of maternal antibodies, virus titres in liver, spleen and mesenteric lymph node were still high on day 5 post infection.

**Conclusions:**

Rabbit kittens are susceptible to infection with very low doses of RHDV, and can transmit virus before they seroconvert. They may therefore play an important role in RHDV field epidemiology, in particular for virus transmission within social groups during virus outbreaks.

## Introduction

Rabbit haemorrhagic disease (RHD) was first described in 1984 and the infectious agent, Rabbit Haemorrhagic Disease Virus (RHDV) was later identified as a member of the *Caliciviridae* family of non-enveloped, positive-strand RNA viruses [1-3]. The virus is highly contagious and kills up to 95% of infected wild and domestic adult European rabbits (*Oryctolagus cuniculus*) causing high losses to wild and farmed rabbits [4-9]. Wild rabbits are an important part of many European ecosystems as well as a popular target for recreational hunting. Domestic rabbits are farmed worldwide for meat and fur [10]. However, in Australia, following the introduction of wild rabbits in 1859, they quickly became and remain one of the worst introduced vertebrate pest species, causing immense losses to the agricultural industries and having a devastating impact on Australia’s biodiversity [9-14].

In 1995, the Czech CAPM-v351 strain of RHDV (herein referred to as RHDV-v351) was unintentionally released in Australia while being evaluated as a biological control agent for rabbits and subsequently deployed to control rabbit populations with enormous initial success [15]. In recent years rabbit populations have recovered [16] potentially due to a combination of high levels of population immunity to RHDV-v351 [17-19], rabbits developing genetic resistance against the virus [20,21] and the presence of a non-pathogenic rabbit calicivirus acting as an imperfect natural vaccine [22,23].

In an effort to complement rabbit control programs in Australia, additional RHDV strains are currently being evaluated to determine their usefulness for release in addition to RHDV-v351. There are indications that members of the RHDVa subgroup of RHDV, which are antigenically distinct to classical RHDV strains like the v351 strain, are replacing the classical RHDV strains in both farmed and wild rabbits in some Asian and European countries [9,24-27]. RHDVa has so far not been described in wild populations from the Iberian Peninsula [28-31]. It is very rare in French rabbit populations [32] but common in Italy where it was mainly described in domestic breeds [33]. Since this suggests that RHDVa strains potentially have a competitive advantage over classical RHDV in the field they may be able to complement existing biological rabbit control in Australia. A possible explanation for the increased competitiveness of RHDVa strains is their reported ability to cause fatal infections in rabbits incompletely immunised with a standard RHDV vaccine [34]. Hence, RHDVa strains might be able to partially overcome immunity to classical RHDV strains in field rabbits by avoiding antibodies to some key epitopes [24]. It is further known that classical RHDV and RHDVa strains have a slightly different specificity for histo-blood group antigens, which may enable RHDVa strains to infect rabbits that are naturally less prone to infection with classical RHDV strains [21,35]. RHDVa strains are hence currently being assessed in adult rabbits to examine their potential to improve rabbit biocontrol in Australia (A.J.R. unpublished).

To most reliably predict the outcome of the release of an additional RHDV strain it is essential to consider as many potential factors influencing RHDV epidemiology as possible and the current knowledge suggests that rabbit kittens could play an important role in RHDV epidemiology. Firstly, kittens are innately more resistant to fatal RHD than adult rabbits [36,37]. At four weeks of age this protection is almost complete, and gradually wanes until they are fully susceptible at approximately 10 weeks. Maternal antibodies can protect kittens from acquiring the RHDV infection, and delay RHDV exposure in the young, but are not responsible for the innate resistance to lethal infection [38]. The protection can be completely reversed by artificial immunosuppression, and differences in the innate immune mechanisms of young rabbits have been suggested as the underlying cause for this resistance [39]. Secondly, virus transmission from 2 week old kittens to their dams has been observed, even though very limited pathology and virus replication was detected in livers from kittens 2–4 weeks of age via immunofluorescence detection of virus protein [40,41]. Thirdly, RHDV genomes were detected via polymerase chain reaction (PCR) in tissues of kittens weeks after infection, and systemic and potentially persistent RHDV infections of young rabbits have been suggested [42], although reactivation of RHDV infection and shedding of infectious virus has not been reported. Lastly, kittens that survive RHDV infection develop antibodies that confer protection from further infection and may become part of an immune breeding population, impacting RHDV epidemiology and rabbit biocontrol [8,38,42,43]. Despite their obvious relevance for field epidemiology, to this date a quantitative assessment of RHDV replication and shedding of RHDV in kittens has not been carried out.

To examine the potential role of rabbit kittens in RHDV epidemiology, we quantitatively monitored infection, seroconversion, replication and excretion of the RHDV-v351 biocontrol strain in kittens over time. To complement the evaluation of RHDVa strains in adult rabbits, we further compared the replication kinetics and the ability to spread to bystander rabbits of RHDV-v351 and a candidate RHDVa strain identified in 2008 in Inchaon, South Korea [26]. While our results indicate that rabbit kittens may play an important role in RHD epidemiology, no significant differences in infection characteristics between the current RHDV-v351 and the RHDVa strain in rabbit kittens were detected.

## Results

### RHDV infection characteristics in rabbit kittens

**Figure 1:**
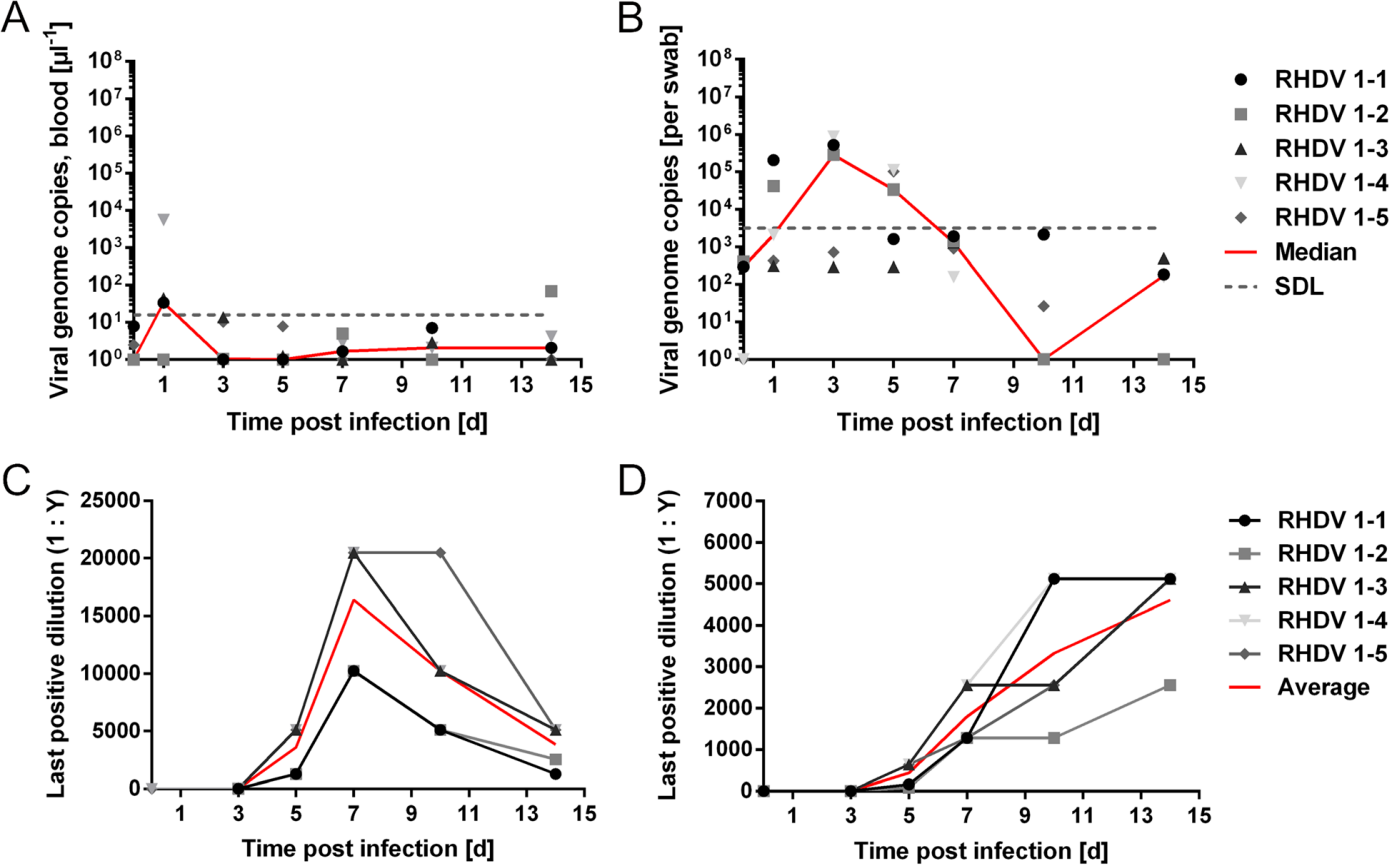
**RHDV replication and excretion in rabbit kittens, which survived and seroconverted.** Rabbit kittens 4–5 weeks of age were infected with 700 ID_50_ of RHDV-v351 as determined in adults and virus replication and shedding was monitored via real-time PCR analysis of virus genome copy numbers in blood and swab samples taken at the indicated times post infection. **A**: Virus genome copy numbers per μl whole blood and **B**: virus genome copy numbers per swab sample. The copy lowest copy numbers that could be accurately detected with the amount of template used in the assay is indicated (SDL = safe detection limit). Serum IgM **(C)** and IgG titres **(D)** were determined in blood samples taken at the indicated times after infection via ELISA as described previously [54,55].

To assess the dynamics of viremia and virus excretion of the current Australian biocontrol strain RHDV-v351, we followed the course of infection over time in five rabbit kittens 4–5 weeks of age infected orally with 700 ID_50_ (as titrated in adult rabbits, A. J. R. unpublished). We monitored virus genome copy numbers in blood and rectal swabs via reverse transcription real-time PCR (RT-qPCR). Median virus genome copy numbers in blood samples were mostly below the detection limit of the assay (16 genomes) and peaked at 1 day post infection (dpi) in three of the five rabbits, and very little viremia was detectable in these kittens 3 dpi and thereafter (Figure 1A). Virus genome copies in rectal swabs peaked between day 1 and 5 and subsequently declined with little virus detectable 10 and 14 dpi (a median of 173 genomes per swab) when kittens were autopsied (Figure 1B). In contrast to the low amounts of virus observed in blood samples at late time points, we detected 350, 800, 14700 18700, 40100 genome copies (average = 15,000) per mg liver at 14 dpi (data not shown). Highest titres of IgM antibodies specific for RHDV were detected at day 7, which slowly decreased until the end of the experiment (day 14, Figure 1C). RHDV-specific IgG was first detected at 5 or 7 dpi and titres increased to day 14 post infection (Figure 1D). No signs of disease were observed during the 14 days the kittens were monitored.

**Figure 2:**
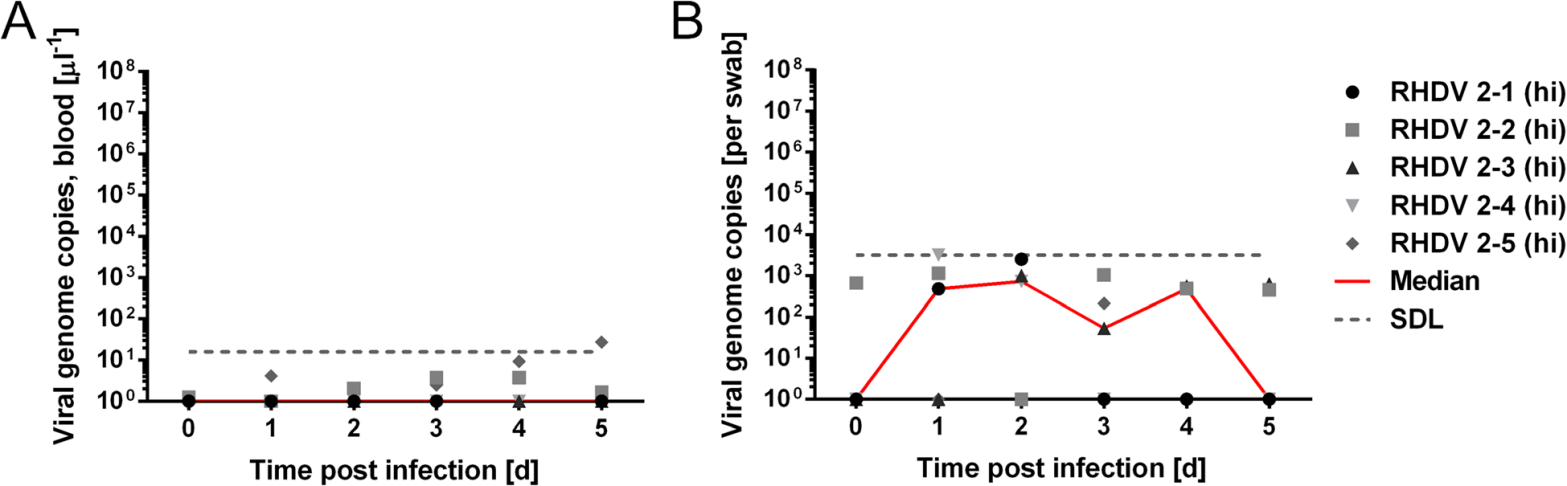
**Virus genome copy numbers in samples from rabbit kittens inoculated with heat inactivated RHDV.** Kittens were inoculated with 1 ml heat-inactivated (hi) RHDV containing 9x10e8 genome copies (equivalent to 2000 ID_50_). Kittens were sampled daily until autopsied at day 5. **A**: Virus genome copy numbers per μl blood; **B**: Genome copy numbers in rectal swab samples. SDL indicates the genome concentration relative to 20 genome copies detected in the RT-qPCR reaction, which is considered the safe detection limit.

To estimate to what extent we detect inoculum or virus progeny due to a productive infection when measuring genome copy numbers, we also inoculated five kittens orally with heat-inactivated virus. Heat-inactivation has been shown to best preserve genome copy numbers measurable by RT-qPCR compared to chemical inactivation procedures [44,45]. RT-qPCR analysis showed a drop of 55% in genome copy numbers in heat-inactivated virus stock compared to untreated virus preparation (9×10^8^ genome copies per millilitre heat-inactivated stock, equivalent to 2000 ID_50_/ml). After inoculation of 1 ml heat-inactivated virus, we measured only trace amounts of virus genomes in daily blood samples (Figure 2A) and in autopsy samples taken at 5 dpi (liver, mesenteric lymph node, spleen, duodenum and bile, data not shown). In contrast, rectal swabs did contain detectable viral genomes with a median of up to 730 genomes per swab between day 1 and 4 (Figure 2B). Therefore, 100-fold higher median viral genome concentrations per swab as measured for untreated virus (compare Figure 1B to Figure 2B) between 1 to 7 dpi, very likely indicate RHDV replication and do not reflect inoculated virus alone. All five kittens inoculated with heat-inactivated virus were serologically negative for RHDV-specific IgM at 5 dpi, indicating that virus replication had not occurred.

In contrast to all previous studies where very high doses (usually >1500 ID_50_) of virus were used to infect kittens, we next examined if kittens are also susceptible to infection with a dose as low as 10 ID_50_ (titrated in adult rabbits). Notably, of the five kittens inoculated, one kitten died at 3 dpi presenting typical RHD liver pathology (discoloured liver, enlarged spleen) at autopsy. The remaining four kittens did not show signs of disease and seroconverted, as indicated by IgM titres ≥ 1:1280 at 5 dpi and IgG titres > 1:20000 at 14 dpi, demonstrating that kittens do not require a substantially higher dose than adults to become productively infected.

### Comparing the currently used biocontrol strain RHDV-v351 with a potential RHDVa candidate for future release

**Figure 3:**
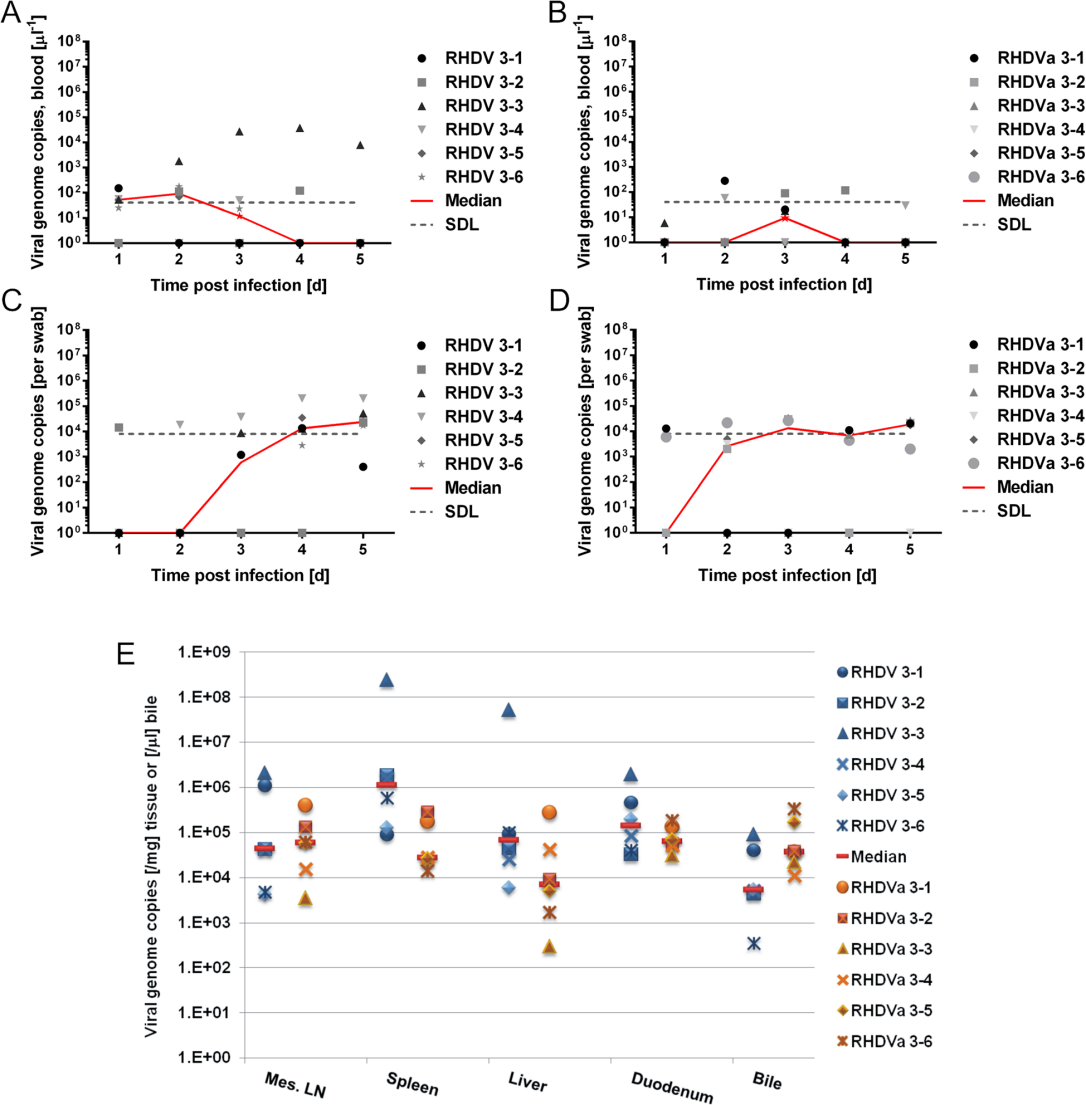
**RHDV and RHDVa replication, excretion and tissue distribution in rabbit kittens.** Shown are virus genome copy numbers per μl whole blood **(A, B)** or rectal swab **(C, D)** as measured via RT-qPCR in samples from RHDV **(A, C)** and RHDVa **(B, D)** infected kittens 4–5 weeks of age at the indicated time points. SDL indicates the genome concentration relative to 20 genome copies detected in the RT-qPCR, which is considered the safe detection limit. **E**: Virus loads in tissues of RHDV and RHDVa infected kittens, 5 dpi. The safe detection limit of our assay in these tissues was 400 genome copies/mg on average (range 140–678 depending on the amount of tissue used for RNA extraction) or 20 genome copies/μl bile.

To compare RHDV-v351 and the potential candidate RHDVa strain, we infected six kittens with 700 ID_50_ of RHDV and in parallel six kittens with a similar amount of RHDVa (approximately 2000 ID_50_ as titrated in adult rabbits). Low levels of replication were observed for the RHDV-v351 strain, with peak genome copy numbers in blood after 2 dpi (Figure 3A), except for one kitten that showed a pronounced viremia until the end of the experiment (day 5). RHDVa genome copy numbers of up to 280 genome copies/μl blood were observed, but median genome concentrations were lower than observed for RHDV (Figure 3B). Genome copy numbers in swab samples were comparable at day 4 and 5 between RHDV and RHDVa (Figure 3C, D). Concentrations of genome copy numbers in mesenteric lymph node, spleen, liver, duodenum and bile did not differ significantly in RHDV and RHDVa infected kittens at autopsy, 5 dpi (Figure 3E), indicating a similar tissue specificity. In line with the low level of virus replication, we only saw signs of RHD at autopsy in one RHDV infected kitten (RHDV 3–3) that had a pale liver.

**Figure 4:**
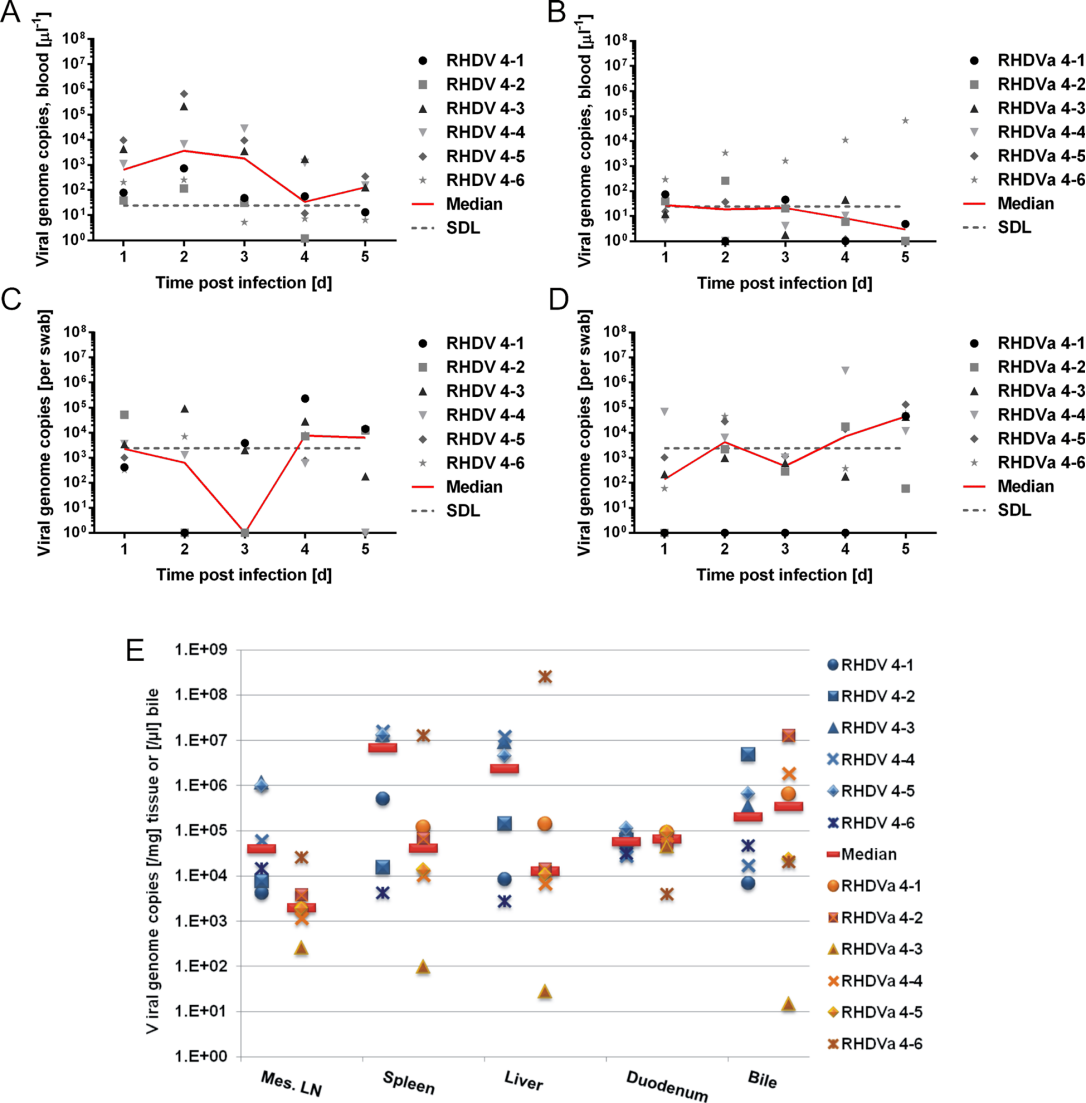
**RHDV and RHDVa replication, excretion and tissue distribution in rabbit kittens infected with a high dose of inoculum.** Shown are virus genome copy numbers per μl whole blood **(A, B)** or rectal swab **(C, D)** measured via RT-qPCR in samples taken from RHDV **(A, C)** and RHDVa **(B, D)** infected kittens 4–5 weeks of age at the indicated time points. SDL indicates the genome concentration relative to 20 genome copies detected in the RT-qPCR, which is considered the safe detection limit. **E**: Virus loads in tissues of RHDV and RHDVa infected kittens, 5 dpi. The safe detection limit of our assay in these tissues was 40 genome copies/mg on average (range: 35–71 depending on the amount of tissue the RNA was extracted from) or 12 genome copies/μl bile.

We repeated these experiments using a higher amount of inoculum equalling 4,300 ID_50_ of RHDV (1.6×10^9^ genomes) and 53,000 ID_50_ of RHDVa (4.7×10^10^ genomes). Inoculation of such high amounts of virus resulted in high viral loads in the blood of two of six RHDV infected animals (2.2×10^5^ and 6.8×10^5^ genome copies/μl) at 2 dpi and a third kitten had 2.8×10^4^ genomes/μl blood at day 3. While the amount of viral genomes mostly decreased in blood samples towards later time points, one RHDVa infected kitten showed increasing viremia from 3 dpi onwards, reaching 7×10^4^ genomes/μl on day 5 (Figure 4A, B). Viral genome numbers measured in rectal swabs revealed a biphasic curve with a first peak between day one and two and another one at day 4–5. Median RHDV genome copy numbers in rectal swabs at day 4 to 5 pi were 2–7 times higher compared to RHDVa (Figure 4C, D). Notably, the >10 times higher dose of virus used to inoculate RHDVa was not reflected in the genome copy numbers measured in rectal swab samples. The amount of virus genomes detected in tissues taken 5 dpi varied considerably between individuals, with very high virus loads observed in the kittens that also had high genome copy numbers in blood. Interestingly, median genome copy numbers in duodenum and bile were very similar between strains (Figure 4E). None of the kittens showed clinical signs of RHD during this experiment, but at autopsy clear evidence of RHD (discoloured liver, enlarged spleen) was observed in two RHDV infected kittens (RHDV 4–3 and RHDV 4–4) and in kitten RHDVa 4–6, which still showed increasing viremia at the time of autopsy. The Korean RHDVa strain reached lower blood virus concentrations compared to RHDV, even when inoculated at a much higher dose. No differences in rectal virus excretion and tissue loads were obvious.

### Comparative transmission studies of RHDV-v351 and RHDVa

**Figure 5:**
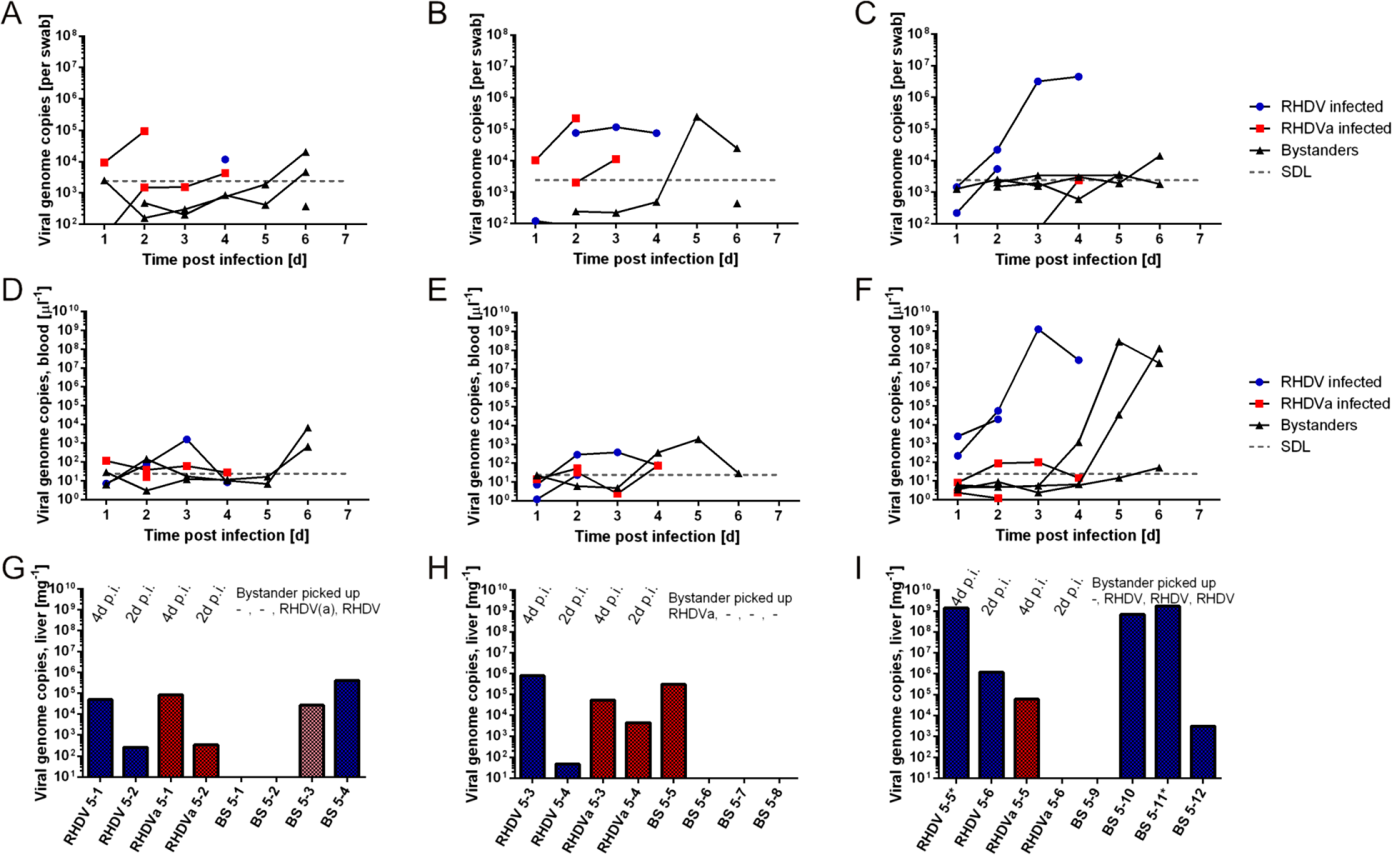
**Infectious RHDV and RHDVa particles are shed from infected kittens and virus is transmitted to littermates.** Two kittens infected with either RHDV or RHDVa (high dose of inoculum) were placed with four uninfected littermates (bystanders, BS). Each column represents one replicate of the experiment done in triplicates (trial 1: **A,D,G**; trial 2: **B,E,H**; trial 3: **C,F,I**). All kittens were monitored for virus replication via blood and swab samples. Experimentally infected kittens were euthanased at 2 or 4 dpi, and bystanders six days after the trial started. Upper panel: Virus shed over time by kittens infected with RHDV (blue) or RHDVa (red) and bystanders (black); Middle panel: Virus concentration in blood samples of infected and bystander kittens (colour code as above). Lower panel: Virus load in livers of infected kittens and bystanders at the time of autopsy, 2 and 4 dpi. Blue and red bars indicate RHDV and RHDVa genome copy numbers, respectively. SDL **(A-F)** indicates the genome concentration relative to 20 genome copies detected in the RT-qPCR, which is considered the safe detection limit The average safe detection limit in the liver tissues was 46 genomes/mg (range 30 – 82). Identification and quantification of the virus that infected the bystanders was done by analysing the liver RNA extracts using a RT-qPCR specific for either RHDV or RHDVa **(G-I)**. In case of one bystander (BS 5–3, see **G**) both PCRs were positive, and a determination of RHDV genome copy numbers with the specific RT-qPCR assay indicated that most (>90 %) of the genomes in its liver were RHDVa genomes (pink bar). Kitten RHDVa 5–6 did not get infected. *indicates kittens that were found dead, both in the morning of days when autopsies were scheduled.

In addition, we examined if transmission of virus to bystander kittens occurs, and if the two strains differ in their transmissibility/infectivity, by group-housing one kitten infected with RHDV and one with RHDVa with four uninfected bystanders. The experiment was carried out in triplicates. Only one of the 12 bystanders became infected with the RHDV-v351 strain, indicated by 13,000 genome copies per mg liver taken at autopsy, 5 days after being housed with the infected kittens (data not shown).In a second transmission study, two kittens infected with the high dose of RHDV and two infected with RHDVa were housed with four uninfected bystanders in one cage, 24 h post infection. Virus replication in the infected animals was monitored daily in blood samples and rectal swabs. The infected animals were euthanized at day 2 (n = 2) and day 4 (n = 2). Bystander rabbits were also monitored daily for virus replication by collecting blood and rectal swabs, and were euthanized at day 5 after their placement with the infected littermates. Livers of all animals were collected at autopsy for virus quantification. This experiment was also carried out in triplicate. Six of the 12 bystander rabbits acquired an infection, shown by detectable RNA in swab, blood and liver samples (Figure 5), demonstrating that kittens can shed infectious virus that is transmitted to littermates. Using PCR assays specific either for the RHDV or RHDVa strain, we determined that four of the six kittens that acquired an infection got infected with the RHDV strain and two with the RHDVa strain. One of the RHDVa infected bystander kittens was also positive for RHDV (with < 10% of detected genome copies being RHDV), indicating a mixed infection. It is worth noting that 3 of the 4 RHDV positive bystanders came from the same cage in which one kitten initially inoculated with RHDV (RHDV 5–5), was found dead at 4 dpi with very high amounts of viral genomes in swab, blood and liver samples (Figure 5C, F, I). One of the three bystanders (BS 5–11) in that cage also was found dead with clear signs of RHD at autopsy and another kitten (BS 5–10) had a high viremia five days after being placed with the infected kittens and a very pale liver at autopsy.

In summary, our results confirm that kittens are susceptible to productive RHDV infection after oral inoculation. RHDV infections in kittens were mostly apathogenic, with little virus detectable in blood and tissues. Virus clearance started at day 3 post infection, at the same time as IgM became detectable. Only a few kittens developed high virus loads and these also displayed signs of disease at autopsy or died. Differences between the RHDV and RHDVa strain were minor and not statistically significant with slightly lower amounts of virus in the blood and less pathogenicity detected for the RHDVa strain.

## Discussion

Our results verify and add to the existing knowledge about RHD in rabbit kittens: It has been shown that kittens can be infected with RHDV if high amounts of virus (at least 1500 LD_50_) are inoculated intra muscularly or orally [38,40,41,46]. We demonstrate here productive infection and seroconversion in 4 of 5 kittens (and one death) after oral inoculation with 10 ID_50,_ which is at least 150-times less than what had been previously used to inoculate kittens. The infectious dose for domestic seronegative rabbit kittens therefore does not appear to be substantially higher than for adult rabbits.

Virus amounts observed in livers of kittens autopsied at 5 dpi were approximately 1000-times lower compared to adult rabbits that succumbed to infection ([47] and A.J.R., unpublished). The only exceptions were the eight out of 82 kittens exposed to the virus that died or showed signs of disease at autopsy and may have died if the trial had been continued. In kittens showing signs of disease, virus loads were higher in liver and blood compared to kittens with subclinical infections, which demonstrates directly for the first time that pathogenicity of RHDV in rabbit kittens is linked to high levels of virus replication. Furthermore, our results indicate that in the small proportion of 4–5 week old rabbits that succumb to infection [38], RHDV does not cause a prolonged disease or a gradual increase of replication levels, but rather shows the same characteristics as observed in adult rabbits. Despite the low levels of replication, a strong adaptive immune response was induced in all kittens tested for seroconversion (5 of 5 kittens infected with 700 ID_50_ RHDV, shown in Figure 1, and 4 of 4 as mentioned in the text infected with 10 ID_50_ of RHDV).

While virus RNA can still be detected in tissues of convalescent rabbits months after primary infection, this RNA has never been demonstrated to be infectious [48,49], and no reactivation of RHDV infections has been described. In our study the amount of virus shed from kittens declined with the onset of the adaptive immune response (Figure 1) and the period of virus excretion was limited to a few days after infection in surviving disease-free kittens. The likelihood that prolonged RHDV infection in kittens may represent a reservoir for RHDV between outbreaks [42,46] hence appears low. This is also supported by a recent genetic study indicating that subsequent annual outbreaks at one site are caused by genetically different RHDV strains [50]. Nevertheless, our results show that kittens can shed virus in sufficient amounts to potentially transmit RHDV in the field, and may therefore play an important role for transmitting the virus during an outbreak, in particular within warren systems. However, the experimental conditions mean that we cannot completely exclude the possibility that some of the virus that infected the bystanders in the transmission experiment was from inoculum shed by the orally infected kittens and not virus progeny resulting from productive infection.

An RHDVa strain with different infection characteristics compared to RHDV-v351 in rabbit kittens could significantly alter RHD epidemiology and the effectiveness of Australian viral rabbit control efforts, e.g. by causing more kittens to seroconvert or by being spread more effectively due to increased replication in kittens. As a case in point, a recently described RHDV variant termed RHDV2 appears to be replacing other field strains of RHDV and RHDVa in wild rabbit populations and rabbitries in Europe [29,51-53]. Notably, this new variant has been reported to kill young rabbits more effectively, further pointing to an important role kittens may play in RHDV epidemiology and highlighting the need to study virus isolates considered for release as additional rabbit control agents in Australia in young rabbits. Hence, we also examined the infection characteristics of an RHDVa strain and showed that fewer kittens developed high virus loads and signs of disease after infection and there were no fatalities compared to infection with RHDV. As the amount of RHDV- v351 and RHDVa used for infection in this trial was not identical, a direct comparison is difficult to make. However, lower genome copy numbers in blood and tissues as well as less virus excretion and transmission were observed even when 12-times higher amounts of RHDVa compared to RHDV were inoculated into kittens (Figures 4 and 5), although this difference was not statistically significant. This suggests that the RHDVa strain might be less virulent in New Zealand White rabbit kittens than the current RHDV biocontrol strain, independent of the amount of virus inoculated.

Previous studies have shown that wild rabbits have an increased level of resistance to infection with RHDV-v351 [20]. It is feasible that the replication kinetics of RHDVa also differs in wild rabbits – this will need to be assessed experimentally in future studies. However, replication and transmission properties in kittens alone are not likely to be the sole determining factor for a putative additional biocontrol strain to be considered for release in Australia. More importantly, virulence parameters in adult rabbits (wild and domestic) such as efficient replication, high mortality rates and infectivity/transmissibility, as well as the ability to partially overcome antibodies to RHDV and the benign calicivirus RCV-A1 will be key to defining a suitable additional RHDV strain able to complement rabbit biocontrol in Australia. A number of candidates are currently being investigated.

## Materials and methods

### Virus strains and preparations

The inoculum of the Czech CAPM-v351 RHDV strain was a commercially available virus preparation used in rabbit biocontrol (RHDV batch 1C, produced at the Elizabeth Macarthur Agricultural Institute (EMAI); the RHDVa inoculum was clarified 10% liver homogenate in PBS of an adult rabbit infected with the RHDVa strain described as 08Q712-1 [26]. Heat-inactivated RHDV stock was generated by incubating the virus preparation in 2 ml screw cap tubes for 180 s at 85°C in a water bath to ensure a drop in infectious virus of more than 99.9% [44,45]. RT-qPCR analysis of the heat inactivated RHDV suspension indicated a drop in detectable genomes to 45% compared to untreated virus suspension. Virus amounts inoculated into rabbits are given as multiples of ID_50_ determined in adult rabbits (older than 12 weeks). The RHDV and RHDVa preparations used had comparable ID_50_ to genome copy ratios.

### Rabbit trials

Animal trials were carried out according to Australian Animal Research Acts and the Australian Code of Practice for the care and use of animals for scientific purposes at the EMAI in Australian Quarantine Inspection Service approved facilities and at the CSIRO Black Mountain site. All procedures were approved by the respective animal ethics committees (M11/09 and M10-09, as well as CESAEC 11–01 and DOMRAB-12). Rabbit kittens were bred from four New Zealand White does, which were not vaccinated against RHDV, and one vaccinated male rabbit in the CSIRO Ecosystem Sciences rabbit breeding colony. The colony is routinely monitored and confirmed free of the benign calicivirus RCV-A1 [23]. Kittens were weaned at 28 days of age and had *ad libitum* access to food (commercial rabbit pellets and autoclaved lucerne hay) and water. Rabbits from the same litter were group-housed during the experiments whenever possible. At 31 days of age (+/−1 d), kittens were infected with RHDV or RHDVa and monitored daily. Blood samples were taken from the marginal ear vein (50 μl) and pipetted into 50 μl distilled water to lyse blood cells. Swab samples were taken with the copped end of Swisspers® cosmetic cotton tips. Excess faecal material was stripped, before the swabs were eluted in 400 μl PBS. To assess seroconversion of kittens infected with RHDV, additional volumes of blood were collected into Vacuettes® (Greiner Bio-One, with clot activator) and the serological status monitored via ELISA (see below). Tissue samples including mesenteric lymph node, spleen, liver and duodenum, as well as bile, were collected during autopsy after euthanasia via intracardiac barbiturate injection following anaesthesia with Zoletil (50 mg/kg). Samples were frozen at −80°C until further processed.

To compare RHDV and RHDVa strains, equal numbers of kittens from different litters were infected with one of the viruses and littermates infected with the same virus were housed together. Transmission experiments were done by placing infected kittens with uninfected littermates 24 hrs post inoculation, to reduce the chance of infecting bystanders due to spillage of inoculum.

### RNA extractions

RNA isolation was done using the Qiagen RNeasy (tissues) and Invitrogen’s PureLink Viral RNA/DNA kit (swab, blood and bile) from samples obtained during the experiments with RHDV alone (Figures 1 and 2). RNA was extracted from 50–200 μl of swab eluates, 50 μl supernatant of the 1:1 blood-water mixtures after centrifugation for 10 min at 13000 g^−1^, 50 μl of bile, and 50 μl of approximately 50 mg tissue lysed in 1 ml PBS via bead beating (glass beads, 50 sec, Mini Bead beater, BioSpec).

For the comparative experiments and transmission studies (Figures 3, 4 and 5), total nucleic acids were extracted from blood, rectal swabs and tissue homogenates using the MagMAX-96 RNA Isolation Kit (Cat AM 1836–5, Ambion, Texas, USA) on a KingFisher™ 96 magnetic particle handling system (Thermo Fisher Scientific, Finland). Lysis-binding buffer (130–200 μl) was placed into a deep well plate (Cat. No. 97040450, Thermo Scientific) followed by sample (50 μl blood, bile and tissue homogenate, up to 200 μl swab eluate). The addition of magnetic beads, washing and elution of nucleic acid followed the manufacturer’s instructions and the manufacturer supplied extraction programs ‘AM1836 DW std’ or ‘AM1836 DW 200v2’ for the KingFisher™ 96 system (for 50 μl or 200 μl sample volume, respectively). Nucleic acids were eluted in 30 or 50 μl H_2_O. Tissue homogenisation was done as described above but using Zirconia beads and a mini bead-beater (Biospec Products, Bartesville, USA).

### Reverse transcription real-time PCR analysis

Standards for reverse transcription-real time PCR were generated from RHDV and RHDVa RNA, using primer 5′ CTC GCC AGT GGT ATT ATA AAT CTT AAC AC-3′ for reverse transcription. The cDNA was amplified using the same primer and 5′ GAC AAC AGG TAA TAC GAC TCA CTA TAG GGA CTG CAA CCA GTA CC-3′, which contains a T7-promoter. The PCR products were purified and used for *in vitro* transcription (Promega, Riboprobe kit) according to the manufacturer’s instructions. RT-qPCR was done using the One-step rt-PCR kit with Sybr Green (Bio-Rad) on a Bio-Rad CFX96/C1000 Thermal cycler platform, using the following primers: 5′-ACC CAG TAC GGC ACR GGC TCC CAA CCA C-3′ and 5′-CTA TCT CCA TGA AAC CAG ATG CAA AGG T-3′ [47], which amplified the respective RHDV and RHDVa fragments with comparable efficiency. Strain-specific PCR analysis was carried out as described above using 5′-GTT GTA TTC GGG CAA CCG GAA G-3′/ 5′-CTA TGG AAC ACA AGC AAA CAT CAC CA-3′ (RHDV) and 5′-GGT TGT TTA TGG TGC CTGTAA AGC A-3′/5′-GCA CAA ATT ACC TTG TCC CAA AAG TTT-3′ (RHDVa).

RT-qPCR assays started with 10 min at 50°C for reverse transcription, followed by 5 min denaturation at 95°C. Cycle conditions were 10 s at 95°C denaturation, 40 s at 63°C annealing and elongation and 10 s at 78°C data acquisition repeated 41 times and followed by a melt curve analysis (65-95°C, 0.5°C increments, 5 s per increment). Reactions with a total reaction volume of 10 μl including 1 μl of the extracted RNA were set-up manually in triplicates in 96-well plates (Bio-Rad, Multiplate™) on ice and the plates sealed with Microseal B Adhesive Sealer (Bio-Rad). For cycler control, data analysis and evaluation the CFX manager 2.0 software (Bio-Rad) was used. The assay has a detection limit of 20 genomes/reaction [47] and the respective threshold for genome copy numbers per mg tissue, swab or μl blood are indicated by a dotted line in the figures or the figure captions. Samples that gave a negative result in the RT-qPCR analysis were set to 1 genome copy for subsequent analysis and illustration.

### Titration of RHDV-specific IgG and IgM in rabbit sera

Serum was separated from blood collected in evacuated tubes (Vacuettes®, Greiner Bio-One) from the marginal ear vein (or from the heart of the anaesthetised animals prior to euthanasia) and subsequently analysed via RHDV IgG and IgM specific ELISA [54] according to the published protocol [55].

### Data analysis tools

Testing for significant differences between the RHDV and the RHDVa strain was done using two-tailed student t-tests or Wilcoxon matched-pairs signed ranked tests (if normal distribution could not be assumed) and GraphPad Prism 6. The most significant difference between the RHDV and RHDVa strain detected was p = 0.065 (exact p-value) for median virus genome copy numbers in blood samples after high dose infection (Figure 4).

## Competing interest

The authors declare that they have no competing interest.

## Authors’ contributions

Designed study: MM, TS, PJK; Carried out experiments: MM, TS, PJK, AJR, SH, JDW, PH; Analysed and interpreted data: MM, TS, PJK; Wrote Manuscript: MM, TS, PJK; contributed reagents/analysis tools: ARJ, PH. All authors read and approved the final manuscript.
